# Phosphotyrosine profiling identifies ephrin receptor A2 as a potential therapeutic target in esophageal squamous‐cell carcinoma

**DOI:** 10.1002/pmic.201400379

**Published:** 2015-01-15

**Authors:** Nazia Syed, Mustafa A. Barbhuiya, Sneha M. Pinto, Raja Sekhar Nirujogi, Santosh Renuse, Keshava K. Datta, Aafaque Ahmad Khan, Kotteazeth Srikumar, T. S. Keshava Prasad, M. Vijaya Kumar, Rekha Vijay Kumar, Aditi Chatterjee, Akhilesh Pandey, Harsha Gowda

**Affiliations:** ^1^ Institute of Bioinformatics, International Technology ParkBangaloreIndia; ^2^ Department of Biochemistry and Molecular BiologyPondicherry UniversityPuducherryIndia; ^3^ Johns Hopkins University School of MedicineBaltimoreMDUSA; ^4^ Adrienne Helis Malvin Research FoundationNew OrleansLAUSA; ^5^ Manipal UniversityManipalIndia; ^6^ Centre of Excellence in BioinformaticsSchool of Life SciencesPondicherry UniversityPuducherryIndia; ^7^ School of BiotechnologyAmrita Vishwa VidyapeethamKollamIndia; ^8^ School of BiotechnologyKIIT UniversityBhubaneswarIndia; ^9^ Department of SurgeryKidwai Memorial Institute of OncologyBangaloreIndia; ^10^ Department of PathologyKidwai Memorial Institute of OncologyBangaloreIndia; ^11^ McKusick‐Nathans Institute of Genetic MedicineJohns Hopkins University School of MedicineBaltimoreMD, USA; ^12^ Department of Biological ChemistryJohns Hopkins University School of MedicineBaltimoreMDUSA; ^13^ Department of OncologyJohns Hopkins University School of MedicineBaltimoreMDUSA; ^14^ Department of PathologyJohns Hopkins University School of MedicineBaltimoreMDUSA

**Keywords:** Biomedicine, In vivo labeling, Mass spectrometry, Post‐translational modifications

## Abstract

Esophageal squamous‐cell carcinoma (ESCC) is one of the most common malignancies in Asia. Currently, surgical resection of early‐stage tumor is the best available treatment. However, most patients present late when surgery is not an option. Data suggest that chemotherapy regimens are inadequate for clinical management of advanced cancer. Targeted therapy has emerged as one of the most promising approaches to treat several malignancies. A prerequisite for developing targeted therapy is prior knowledge of proteins and pathways that drive proliferation in malignancies. We carried out phosphotyrosine profiling across four different ESCC cell lines and compared it to non‐neoplastic Het‐1A cell line to identify activated tyrosine kinase signaling pathways in ESCC. A total of 278 unique phosphopeptides were identified across these cell lines. This included several tyrosine kinases and their substrates that were hyperphosphorylated in ESCC. Ephrin receptor A2 (EPHA2), a receptor tyrosine kinase, was hyperphosphorylated in all the ESCC cell lines used in the study. EPHA2 is reported to be oncogenic in several cancers and is also known to promote metastasis. Immunohistochemistry‐based studies have revealed EPHA2 is overexpressed in nearly 50% of ESCC. We demonstrated EPHA2 as a potential therapeutic target in ESCC by carrying out siRNA‐based knockdown studies. Knockdown of EPHA2 in ESCC cell line TE8 resulted in significant decrease in cell proliferation and invasion, suggesting it is a promising therapeutic target in ESCC that warrants further evaluation.

**Abbreviations**
EPHA2: ephrin receptor A2
ESCC: esophageal squamous‐cell carcinoma

## 1 Introduction

Esophageal cancer is one of the leading causes of cancer‐related mortality, which annually affects more than 450 000 people worldwide 1. Esophageal carcinoma occurs in two major forms: esophageal adenocarcinoma and esophageal squamous‐cell carcinoma (ESCC). The incidence of adenocarcinoma and squamous‐cell carcinoma varies widely by region. The incidence rate of ESCC is particularly high in Asian countries and is reported to be more than 100 cases per 100 000 annually 1, 2. Tobacco usage and alcohol consumption are some of the established risk factors for ESCC. Esophagectomy is the primary treatment option for locally advanced disease while chemotherapy and radiation have remained a mainstay for treating metastatic and recurrent disease. The overall 5‐year survival of patients with esophageal cancer ranges from 15 to 25%, highlighting the need for effective treatment strategies 1. Early diagnostic markers and novel therapeutic targets can significantly improve clinical management of ESCC. We have carried out gene expression profiling and quantitative proteomics studies that have revealed several biomarkers in ESCC 3, 4, 5, 6.

Last decade has witnessed a paradigm shift in cancer treatment. Cytotoxic chemotherapeutics had remained a mainstay of cancer therapy for several decades. This has changed after the tremendous success of imatinib, a small‐molecule kinase inhibitor used in the treatment of BCR‐ABL‐positive chronic myelogenous leukemia and acute lymphoblastic leukemia 7. This new targeted therapeutic approach relies on inhibiting specific protein or pathway on which the cancer cells are dependent for their survival and proliferation. A number of targeted therapeutic agents are now in clinical use to treat various cancers including metastatic melanoma 8, metastatic breast cancer 9, metastatic renal cancer 10, and non‐small cell lung cancer 11. Cancer genome sequencing efforts have revealed a number of genes that are frequently mutated in various malignancies. One of the challenges associated with data from cancer genome sequencing is the difficulty associated with choosing potentially druggable targets from a large number of mutated genes. Genome sequencing in ESCC has also revealed mutations in a number of protein coding genes. The challenge now is to determine which of these are activating mutations that could be driving proliferation in ESCC 12, 13, 14, 15 and provide us a chance to identify potential drug targets in ESCC.

Aberrant activation of kinase signaling pathways is commonly observed in several malignancies. Some of this is now established to be due to activating mutations in kinases. Thus, kinases have emerged as attractive candidates for targeted therapy in a number of cancers. Kinases regulate most cellular processes through reversible phosphorylation. Therefore, phosphorylation serves as a surrogate for activation of kinase signaling pathways. Phosphoproteomics offers a unique opportunity to study signaling in an unbiased manner. It offers a functional readout in biological systems reflecting proteins and pathways that are activated. We and others have used this approach to characterize aberrant kinase signaling in various cancers and demonstrated its utility in identifying potential therapeutic targets 16, 17, 18. In this study, we carried out phosphoproteomic profiling of ESCC cell lines to determine activated tyrosine kinase signaling pathways. We found hyperphosphorylation of ephrin receptor A2 (EPHA2) in all the ESCC cell lines profiled in this study (TE1, TE2, TE5, and TE8). Using RNAi‐based knockdown experiments, we demonstrate that EPHA2 is a potential therapeutic target in ESCC.

## 2 Materials and methods

### 2.1 Cell lines and culture methods

Non‐neoplastic cell line Het‐1A was procured from the American Type Culture Collection (Catalog CRL‐2692, ATCC, Manassas, VA, USA). All cell lines were grown in humidified incubator at 37°C with 5% CO_2_. Het‐1A was grown on Corning Cell BIND flasks using keratinocyte serum‐free media (KFSM) with human recombinant epidermal growth factor (EGF 1–53), bovine pituitary extract, calcium chloride and 1% penicillin/streptomycin without any serum supplement. The ESCC cell lines TE1, TE2, TE5, and TE8 were cultured using DMEM high‐glucose media, supplemented with 10% FBS and 1% penicillin/streptomycin. All cell lines were serum‐starved for 12 h before harvesting. TE1 is well differentiated; TE8 is moderately differentiated while TE2 and TE5 are poorly differentiated.

### 2.2 Preparation of super‐SILAC mix

For preparation of super‐SILAC mix, two ESCC cell lines (TE2, TE8) were cultured in DMEM high‐glucose media containing ^13^C_6_ arginine and ^13^C_6_ lysine supplemented with 10% FBS. Equal amounts of heavy lysate from these two cell lines were pooled together to create super‐SILAC mix. Super‐SILAC mix was spiked into lysates from Het‐1A TE1, TE2, TE5, and TE8 at 1:5 ratio.

### 2.3 Lysate preparation

Cells were harvested in urea lysis buffer containing 9 M urea, 20 mM HEPES, 1 mM sodium orthovanadate, 1 mMβ‐glycerophosphate, and 2.5 mM sodium pyrophosphate. The protein lysates were sonicated and then centrifuged at 4000 rpm for 20 min and supernatant was collected. Protein estimation was carried out using BCA method. Super‐SILAC mix was used as an internal standard by spiking it in 1:5 ratio into all the cell lysates (5 mg super‐SILAC mix into 20 mg lysate).

### 2.4 In‐solution digestion

All the lysates were digested in‐solution using trypsin. Briefly, lysates were incubated with 5 mM DTT at 60°C for 45 min, followed by alkylation with 20 mM iodoacetamide in dark at room temperature for 15 min. Lysates were diluted using IAP buffer to reduce urea concentration to less than 2 M. Trypsin (sequencing grade trypsin, Promega) was added to all the lysates to achieve 1:20, w/w, enzyme‐to‐substrate ratio. Digestion was carried out overnight at 37°C. The digested peptides were acidified using 1% TFA and centrifuged at 13 000 rpm for 10 min to clear the lysates. Peptide digests were desalted using Sep‐Pak C18 (Waters corporation, Milford, MA, USA) cartridges and lyophilized for 72 h.

### 2.5 Phosphopeptide enrichment

The lyophilized peptides were subjected to phosphotyrosine enrichment using PhosphoScan Kit (Cell Signaling Technology, Danvers, MA, USA) according to the manufacturer's protocol. Briefly, the peptides were incubated with pY100 antibody beads at 4°C for 2 h. The beads were washed in IAP buffer and phosphopeptides were eluted with 0.1% TFA. The eluted peptides were desalted using C18 stage tips.

### 2.6 LC‐MS/MS analysis

The samples were analyzed on LTQ‐Orbitrap Velos mass spectrometer (Thermo Scientific, Bremen, Germany) interfaced with Easy nano‐LC (Thermo Scientific, Bremen, GmbH). The peptides were loaded onto a trap column (2 cm × 75 μm) that was packed with C18 material (Magic C_18_ AQ, 5 μm, 100 Å) with a flow rate of 3 μL/min using 0.1% formic acid and separated on an analytical column (10 cm × 75 μm, Magic C_18_ AQ 3 μm, 120 Å) with a flow rate of 350 nL/min with a linear gradient of 5 to 60% solvent B (90% ACN, 0.1% formic acid) over a period of 100 min. Precursor ions were acquired in *m*/*z* range of 350–1800 with a resolution of 60 000 at 400 *m*/*z*. From each survey scan, 15 most intense ions were selected and fragmented in HCD mode with normalized collision energy of 39. The MS/MS scans were performed at a resolution of 15 000 at 400 *m*/*z*. Capillary voltage was maintained at 2 kV. For internal mass calibration, lock mass option was enabled with polysiloxane ion (*m*/*z*, 445.120025) from ambient air.

### 2.7 Data analysis and quantitation of phosphopeptides

The raw data were searched using MASCOT and SEQUEST search algorithms through Proteome Discoverer (Version 1.4) software (Thermo Fisher Scientific, Bremen, Germany) against NCBI RefSeq release 59 human protein database containing 33 833 sequences and known contaminants. The MS and MS/MS mass error tolerance was set at 20 ppm and 0.1 Da, respectively. The search parameters included carbamidomethylation of cysteine residues as fixed modification, oxidation of methionine, phosphorylation on tyrosine, serine and threonine and heavy lysine (_13_C^6^) and arginine (_13_C^6^) as variable modifications. Trypsin was specified as the protease with a maximum of two missed cleavages. Precursor ion quantitation node was added in the workflow for SILAC quantitation and PhosphoRS node (Version 3.1) was added to determine the probability of phosphorylation at a given residue in a peptide. PhosphoRS probability score >75.0 was used as a threshold for phosphosite identification. Phosphopeptides were mapped to corresponding protein sequences to locate the position of phosphosites in the protein. Phosphopeptides identified with 1% FDR threshold after carrying out decoy database searches were considered for reporting. The ratios for phosphopeptides were calculated by using spiked‐in internal standard as a reference. Across all the five cell lines used in the study, light/heavy ratio was calculated for each phosphopeptide with a corresponding coeluting peak from the spiked‐in internal standard. Phopshopeptide ratio obtained in each cancer cell line was divided by the ratio obtained for corresponding peptide in Het‐1A. This ratio of ratios was used to determine differentially phosphorylated proteins in ESCC. Phosphopeptides that showed increased phosphorylation in cancer cell lines compared to Het‐1A were inferred as hyperphosphorylated.

### 2.8 Western blotting

Cells were harvested and lysed in RIPA buffer (50 mm Tris‐HCl, pH 7.4, 150 mm NaCl, 1 mm EDTA, 1% NP‐40, 0.5% SDS, 0.25% sodium deoxycholate, and 1 mm sodium orthovanadate in the presence of protease inhibitors). Equal amount of whole‐cell lysates of TE1, TE2, TE5, and TE8 were separated on NuPAGE Novex Bis‐Tris 4–12% gradient gels (Invitrogen, Grand Island, NY, USA). Proteins were transferred to nitrocellulose membrane, and probed with anti‐EPHA2 and anti‐phosphoEPHA2 antibodies (Cell Signaling Technology).

### 2.9 siRNA‐based knockdown of EPHA2

Five nanomolar siRNA targeting EPHA2 (Flexitube siRNA Hs EPHA2, QIAGEN, Valencia, CA, USA) and scrambled siRNA (All stars negative control siRNA, QIAGEN) were used for transfection of TE8 cell line with Lipofectamine RNAiMAX (Invitrogen). In brief, half a million cells were transfected with siRNA and cells were harvested 48 h post transfection for assessing knockdown efficiency or other follow‐up experiments.

### 2.10 Cell proliferation assay

Transfected cells were trypsinized and 5000 cells were seeded onto four wells of 24‐well plates in triplicate and stained with 0.5% crystal violet. The stain was eluted with 0.1% acetic acid and absorbance was measured in spectrophotometer.

### 2.11 Invasion assay

Transfected cells were trypsinized and 5 × 10^4^ cells seeded onto Biocoat Matrigel Invasion Chambers (BD Biosciences) in 1% FBS in DMEM high‐glucose medium. Ten percent FBS in DMEM high‐glucose medium was added in the lower chamber as the chemo attractant. The filter membranes were stained with DAPI (Invitrogen) after carefully removing the cells from the inner side and the number of cells that penetrated through the matrigel and membrane was counted in ten randomly selected view fields at 20X magnification.

## 3 Results and discussion

### 3.1 Phosphotyrosine profiling of ESCC cell lines

**Figure 1:**
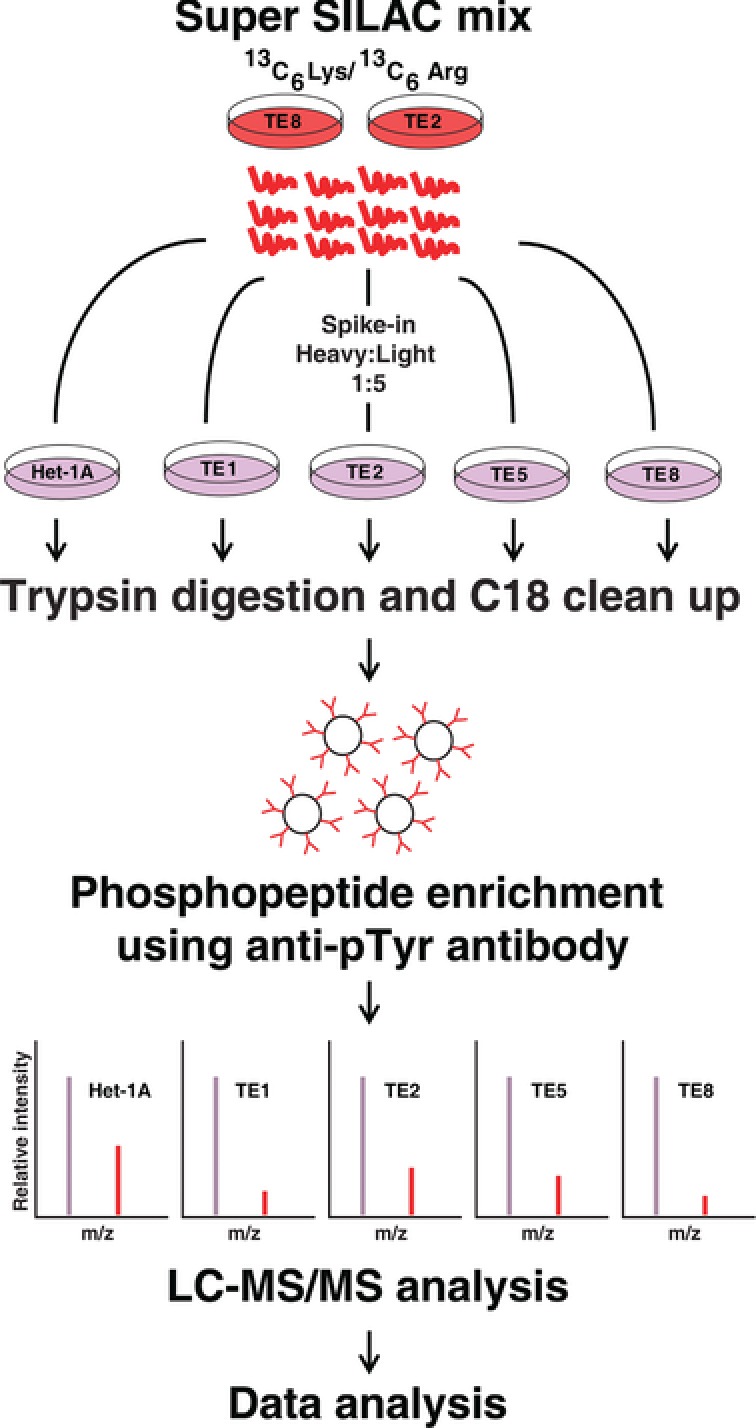
Workflow employed for characterizing tyrosine phosphorylation pattern in ESCC cell lines. A super‐SILAC mix generated by pooling lysates from ^13^C_6_ lysine and ^13^C_6_ arginine labeled TE2 and TE8 cell lines was spiked at 1:5 ratio into all the ESCC cell lines as well as non‐neoplastic cell line Het‐1A. Protein lysates were digested using trypsin and tyrosine phosphopeptides were enriched using anti‐phosphotyrosine antibody. Phosphopeptides were quantitated using spiked‐in internal standard.

**Figure 2:**
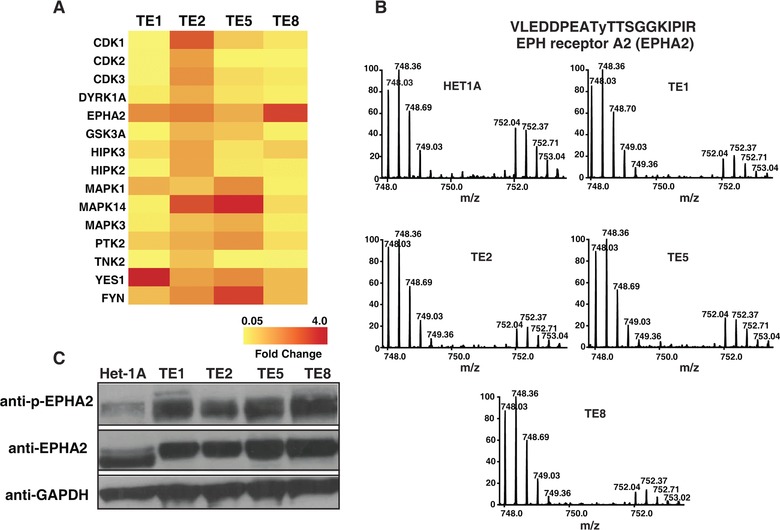
EPHA2 is hyperphosphorylated in ESCC cell lines. (A) A heat map representing relative phosphorylation levels of candidate molecules across four ESCC cell lines compared to Het‐1A. (B) MS spectra showing relative difference in the abundance of EphA2 phosphopeptide (VLEDDPEAT**y**TTSGGKIPIR) across all the cell lines used in the study. Mass‐to‐charge ratio 748.03 represents unlabeled version of phosphopeptide from cell lysates while 752.04 represents SILAC‐labeled version from super‐SILAC mix.

We employed super‐SILAC‐based 19 approach to identify differentially phosphorylated proteins between non‐neoplastic esophageal squamous‐cell line Het‐1A and a panel of ESCC cell lines (Fig. 1). Super‐SILAC mix prepared by pooling lysates from two different ESCC cell lines were spiked into cell lysate from Het‐1A TE1, TE2, TE5, and TE8. The lysates were digested using trypsin and tyrosine phosphopeptides were enriched using anti‐phosphotyrosine antibody based strategy 20. The enriched phosphopeptides were analyzed by LC‐MS/MS. We identified 278 unique phosphopeptides across these cell lines. The list of phosphopeptides identified is provided in Supporting Information Table 1. As expected, protein phosphorylation pattern was heterogeneous across ESCC cell lines (Fig. 2A). EPHA2, a receptor tyrosine kinase, showed consistent hyperphosphorylation across all ESCC cell lines used in the study. Maximum phosphorylation was observed in TE8 where it was found to be fourfold hyperphosphorylated compared to non‐neoplastic cell line Het‐1A (Fig. 2A and B). Overexpression and hyperphosphorylation of EPHA2 was verified by immunoprecipitation and Western blotting experiments (Fig. 2C). Other proteins that showed hyperphosphorylation in ESCC cell lines include catenin (cadherin‐associated protein) delta 1 (CTTNND1); SHC‐transforming (Src homology 2 domain‐containing) protein 1 (SHC1); Src homology 2 domain‐containing adaptor protein B (SHB); neural precursor cell expressed, developmentally downregulated 9 (NEDD9); mitogen‐activated protein kinase 14 (MAPK14); and YES proto‐oncogene 1, Src family tyrosine kinase (YES1). YES1 was significantly hyperphosphorylated in TE1. YES1 is a member of the src family of proteins. YES1 facilitates nuclear translocation of epidermal growth factor receptor 21 and is known to regulate oncogenic pathways in colon cancer progression 22 and non‐small cell lung carcinoma 23. MAPK14 was significantly hyperphosphorylated in TE2. MAPK14 is a member of the MAP kinase family and is overexpressed in various cancers including prostate cancer, head and neck squamous carcinoma, and breast cancer. Overexpression and/or hyperactivation of these tyrosine kinases are frequently observed in several cancers. Our data reveal a similar pattern in ESCC.

### 3.2 EPHA2 and associated signaling components are hyperphosphorylated in ESCC

**Figure 3:**
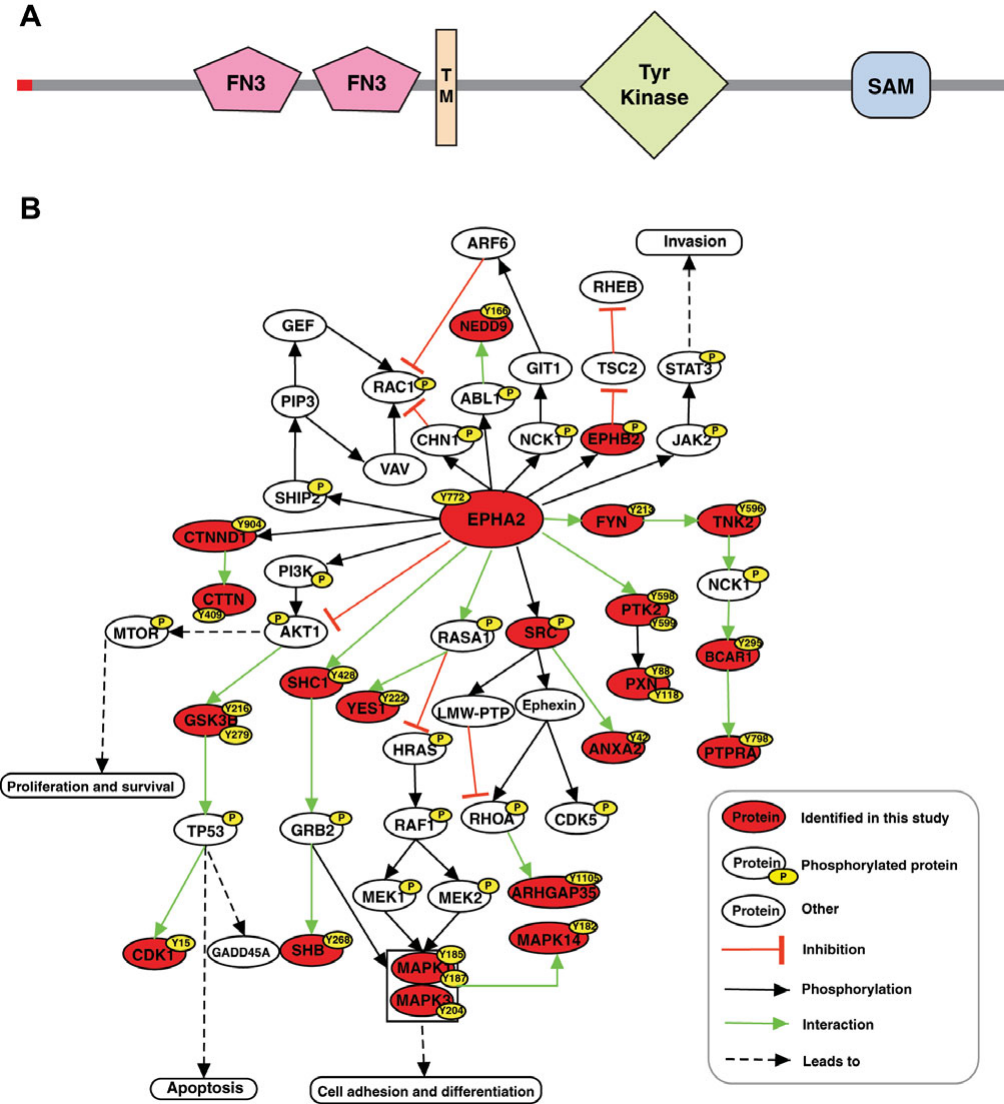
Differential phosphorylation of EPHA2 pathway mediators. (A) Schematic showing domain architecture of EPHA2. (B) EphA2 pathway and various cellular processes that it regulates. Red color marks proteins identified in this study. Phosphorylation sites identified in the study are shown in yellow.

EPHA2 belongs to the ephrin receptor subfamily of protein‐tyrosine kinases 24. It is a 130‐kDa protein that has an extracellular region containing two fibronectin type III repeats, a transmembrane domain and a cytosolic region with a protein tyrosine kinase catalytic domain and a sterile alpha motif domain in the C‐terminus (Fig. 3A). It was earlier named as epithelial cell kinase as it showed high expression in tissues containing epithelial cells 25. Several studies have demonstrated the role of EPHA2 and its endogenous ligand ephrin A1 in angiogenesis and neovascularization 26, 27 and endothelial cell migration and vascular assembly 28. Signaling cascades downstream of EPHA2 involve interaction of cytoplasmic domain with several proteins including p85 subunit of phosphatidylinositol 3‐kinase 29, Src‐like‐adaptor (SLA) 30, vav2 guanine nucleotide exchange factor (VAV2), and vav3 guanine nucleotide exchange factor (VAV3) 31. In our study, we observed differential phosphorylation of several proteins associated with EPHA2 signaling (Fig. 3B). Notable effectors associated with EPHA2 signaling included SRC 32, 33, Src homology 2 domain containing transforming protein 1 (SHC1) 34, mitogen‐activated protein kinase 1 (MAPK1) 34, mitogen‐activated protein kinase 3 (MAPK3), cyclin‐dependent kinase 5 (CDK5) 35, and paxillin (PXN) 36.

### 3.3 Silencing EPHA2 inhibits cell proliferation, invasion, and migration

**Figure 4:**
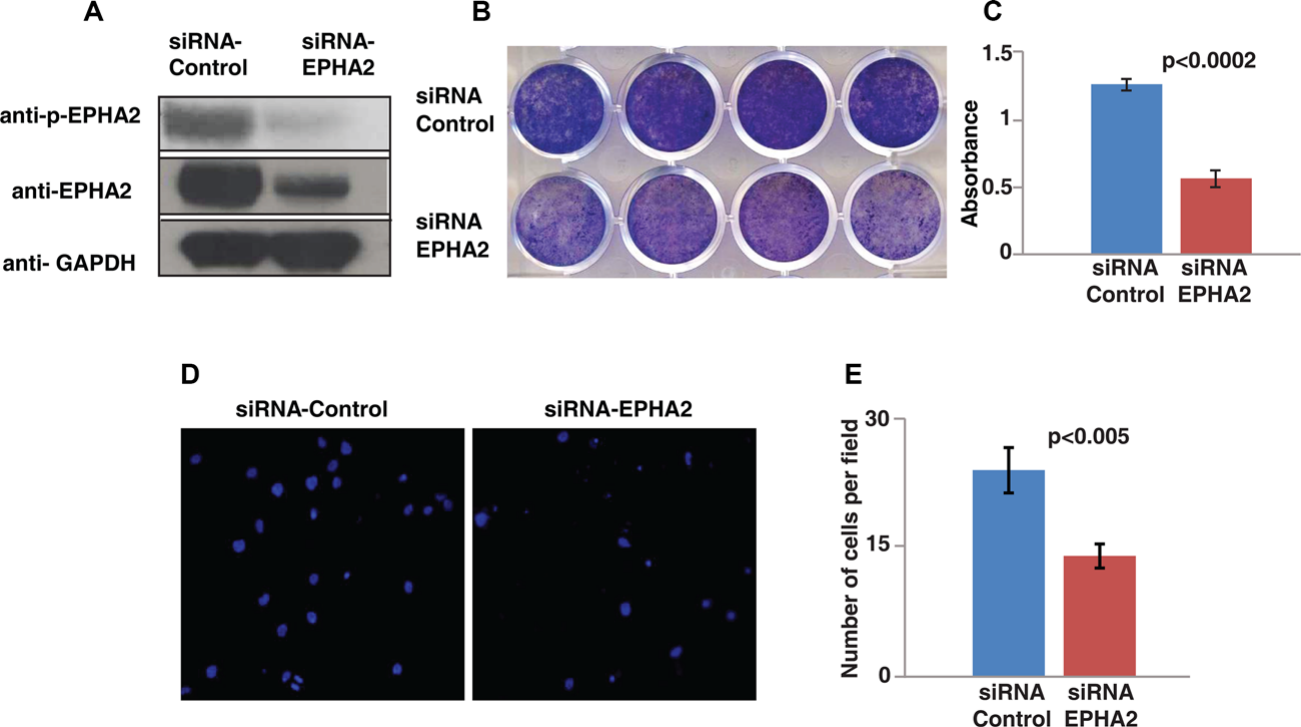
EphA2 knockdown affects cell proliferation and invasion capability. (A) Immunoblotting showing p‐EphA2 and EphA2 protein levels in scrambled siRNA‐ and EphA2‐siRNA‐transfected TE8 cells. (B) Crystal violet staining of TE8 cells transfected with scrambled siRNA and EphA2‐siRNA. (C) Bar graph quantifying absorbance of dissolved dye in the wells shown in (B). (D) A representative microscopic field showing TE8 cells transfected with scrambled siRNA and EphA2‐siRNA that invaded matrigel invasion chambers. (E) Bar graph representing number of cells per field that penetrated through the matrigel.

Immunohistochemical staining in esophageal cancers has revealed that nearly 50% of the tumors display EPHA2 overexpression 37, 38, 39. These studies have also revealed that EPHA2 expression is associated with lymph node metastasis and is a significant predictor of overall survival. These evidences suggest that EPHA2 plays a central role in a subset of ESCC. We reasoned that in cell lines that show overexpression and hyperphosphorylation of EPHA2, inhibition using a pharmacological inhibitor or genetic knockdown should affect their proliferation and invasion capabilities. We carried out siRNA‐mediated knockdown of EPHA2 in TE8 cell line, as it showed maximum phosphorylation among all the cell lines used in our MS‐based screening. siRNA‐mediated knockdown of EPHA2 expression in TE8 cell line significantly decreased cell proliferation (Fig. 4A–C, *p* < 0.0002) and invasion/migration capability (Fig. 4D and E, < 0.005). These studies demonstrate that inhibition of EPHA2 is an effective strategy in ESCC. This warrants genetic and pharmacological inhibition studies in preclinical models of ESCC.

### 3.4 EPHA2 is frequently overexpressed and activated in several malignancies

Phosphoproteomic approach is an effective strategy to investigate signaling pathways in biological systems. We and others have demonstrated the utility of phosphoproteomics approaches in identifying aberrantly activated kinase signaling pathways in various malignancies. Using similar strategy, we found EPHA2 to be consistently hyperphosphorylated in all ESCC cell lines used in our study. Genetic and pharmacological inhibition of EPHA2 in cell culture as well as mouse xenograft‐based studies has demonstrated it as a potential target in various cancers including NSCLC 40. EPHA2 overexpression is reported in a number of cancers including gliomas 41, urinary bladder cancer 42, non‐small cell lung cancers 43, renal cancer 44, esophageal cancer 37, and colorectal cancer 45. In ovarian cancer, EPHA2 overexpression is reported to be significantly and independently associated with poor patient survival 46, 47, 48. In esophageal cancer, patients with EPHA2 overexpression are known to have a poor prognosis compared to those who do not show overexpression 37. In prostate cancers, progressively higher levels of EPHA2 was observed in high‐grade prostatic intraepithelial neoplasia and prostatic carcinoma cells suggesting increased expression of EPHA2 is associated with a more aggressive phenotype 49. Differential EPHA2 epitope display has been observed in malignant cells compared to normal cells suggesting potential new opportunities for therapeutic targeting 50. In non‐small cell lung cancers, increased expression of EPHA2 is observed in smokers and is a predictor of poor survival 51. EPHA2 receptor antagonists have been investigated as potential anticancer therapies to block EPHA2 mediated tumor neovascularization 52, 53. Previous studies have demonstrated that there is a significant correlation between EPHA2 expression and regional lymph node metastasis and number of lymph‐node metastasis 37. In addition, it was reported that patients with EPHA2 overexpression in their tumors have poorer prognosis. Collectively, these data suggest that targeting EPHA2 should be a useful strategy in ESCC.

*The authors have declared no conflict of interest*

## Supporting information

As a service to our authors and readers, this journal provides supporting information supplied by the authors. Such materials are peer reviewed and may be re‐organized for online delivery, but are not copy‐edited or typeset. Technical support issues arising from supporting information (other than missing files) should be addressed to the authors.

**Table S1**. List of phosphopeptides identified in cell lines used in the study
